# RNA-seq and integrated network analysis reveals the hub genes and key pathway of paclitaxel inhibition on Adriamycin resistant diffuse large B cell lymphoma cells

**DOI:** 10.1080/21655979.2022.2048772

**Published:** 2022-03-09

**Authors:** Haoyuan Hong, Bin Luo, Yingying Qin, Sizhu Li, Zhigang Peng

**Affiliations:** a Department of Medical Oncology, First Affiliated Hospital of Guangxi Medical University, Nanning, Guangxi Zhuang Autonomous Region, China; b Department of Hematology, People’s Hospital of Guangxi Zhuang Autonomous Region, Nanning, Guangxi Zhuang Autonomous Region, China; c Department of Gastroenterology, First Affiliated Hospital of Guangxi Medical University, Nanning, Guangxi Zhuang Autonomous Region, China

**Keywords:** Diffuse large B-cell lymphoma, Adriamycin resistant cell line, Paclitaxel, RNA-seq, bioinformatics

## Abstract

About 40% of patients with diffuse large B-cell lymphoma (DLBCL) develop drug resistance after first-line chemotherapy, which remains a major cause of morbidity and mortality. The emergence of DLBCL drug resistance is mainly related to Adriamycin. Our previous research shows that Paclitaxel could be a potential therapeutic drug for the treatment of Adriamycin-resistant DLBCL. Based on the results of RNA-seq and integrated network analysis, we study the potential molecular mechanism of Paclitaxel in the treatment of Adriamycin-resistant DLBCL in multiple dimensions. A CCK-8 assay showed that the inhibitory effect of Paclitaxel on Pfeiffer and Pfeiffer/ADM (Adriamycin-resistant DLBCL cell lines) is significantly higher than that of Adriamycin (*P* ＜ 0.05). Five hub genes (UBC, TSR1, WDR46, HSP90AA1, and NOP56) were obtained via network analysis from 971 differentially expressed genes (DEGs) based on the RNA-seq of Paclitaxel-intervened Pfeiffer/ADM. The results of the network function module analysis showed that the inhibition of Pfeiffer/ADM by Paclitaxel was closely related to ribosome biosynthesis in eukaryotes. The results of RT-qPCR showed that the mRNA levels of the five hub genes in the Pfeiffer/ADM group were significantly lower than those in the Pfeiffer group and the Pfeiffer/ADM Paclitaxel-treated group (*P* ＜ 0.05). Consistent with studies, Paclitaxel exhibited a significant inhibitory effect on Adriamycin-resistant DLBCL, which may have played a role in the five hub genes (UBC, TSR1, WDR46, HSP90AA1 and NOP56) and ribosome biosynthesis in eukaryotes pathway, but the specific regulation needs further experimental verification.

## Introduction

Diffuse large B-cell lymphoma (DLBCL) is the most common type of non- Hodgkin’s lymphoma (about 30%-40%), with a high invasiveness and heterogeneity [1,2]. Currently, the first-line treatment regimen for DLBCL patients is R-CHOP (rituximab, cyclophosphamide, Adriamycin, vincristine, and prednisone). However, there is still a significant number of DLBCL patients whose treatment progress is limited by drug resistance [3–5]. The emergence of DLBCL drug resistance is mainly related to Adriamycin, which is an anthracycline drug used in the first-line chemotherapy regimen of DLBCL [6]. The clinical promotion and application of other treatments of Adriamycin-resistant DLBCL, including immunotherapy and new molecular targeted drugs, are seriously limited because of their potential side effects and high price, resulting in blocked progress of the clinical treatment of Adriamycin-resistant DLBCL. Furthermore, the research and development of new drugs, including drawbacks such as long clinical trials, potential side effects and high prices, have severely restricted the treatment of patients with DLBCL drug resistance [7–10]. Therefore, developing a drug de novo is a laborious and costly endeavor. Thus, the repositioning of already approved drugs for the treatment of Adriamycin-resistant DLBCL is promising and valuable [11].

Paclitaxel, also known as Taxol, is a taxane drug isolated from the bark of Taxus mairei [12]. Following its discovery, Paclitaxel was approved by the US Food and Drug Administration for the treatment of advanced ovarian cancer in 1992 [13]. Currently, Paclitaxel can be used as a single chemotherapy drug or in combination with other drugs in the treatment of ovarian cancer, breast cancer, gastric cancer, non-small cell lung cancer, and other solid tumors [14]. With its characteristics of high efficiency and good safety, Paclitaxel has become one of the most successful and widely used natural anticancer drugs [15].

Some clinical research shows that Paclitaxel has a certain level of efficacy in the treatment of recurrent Adriamycin-resistant DLBCL. For example, the disease remission rate can reach 45% with the weekly use of low-dose Paclitaxel in the treatment of recurrent drug-resistant DLBCL [16]. A phase-II clinical study confirmed that with a TTR regimen (Paclitaxel, topotecan and rituximab) in the treatment of relapsed refractory DLBCL, the 3-year total remission rate was 69% [17]. Paclitaxel exhibits the characteristics of high efficacy and low toxicity in the treatment of Adriamycin-resistant DLBCL. Similarly, in vitro experimental studies have also revealed that Paclitaxel has a significant inhibitory effect on the proliferation of Adriamycin-resistant DLBCL cell lines. For instance, Adriamycin-resistant cell lines were sensitive to Paclitaxel and its derivative docetaxel, and the inhibition rate of Adriamycin-resistant cell lines greatly increased after treatment with Paclitaxel or docetaxel [18]. In clinical practice, we found that Paclitaxel was effective in the treatment of patients with Adriamycin-resistant DLBCL, thus serving as a treatment after the third line [19]. However, its specific molecular mechanism has not been explored. Our previous studies found that Paclitaxel can be used as a potential drug for the treatment of DLBCL, but the inhibition of Paclitaxel on Adriamycin-resistant DLBCL remains unclear [20]. In this study, RNA-seq technology and bioinformatics were used to explore the potential molecular mechanism of Paclitaxel inhibition on Adriamycin-resistant DLBCL cell lines.

## Materials and methods

### Cell culture

The human DLBCL cell line (Pfeiffer) and the Adriamycin-resistant cell line (Pfeiffer/ADM) were generously donated by Professor Cen Hong (Affiliated Tumor Hospital of Guangxi Medical University) [21]. The cells were cultured in an RPMI-1640 medium (Gibco, NY, USA) containing 10% fetal bovine serum (Gibco, NY, USA) and a 1% penicillamine mixture (Solarbio, Beijing, China) in an incubator set at 37°C and supplied with 5% CO_2_. Paclitaxel and Adriamycin were purchased from MedChemExpress Company (MCE, NJ, USA).

### Cell viability assay

The biological effects of Adriamycin and Paclitaxel on Pfeiffer and Pfeiffer/ADM cells were quantified by the cell counting kit-8 (CCK-8) assay. The cells were planted into 96-well plates (density of 5 × 10^4^ cells/well), treated with different concentrations of Adriamycin and Paclitaxel, and incubated for 24, 48, and 72 hours, A 10-μl reagent of CCK-8 (Donjindo, Japan) was added to each well and mixed for 2 hours continuously. Finally, the absorbance value at 450 nm was measured by a microplate reader (Bio-Rad, Hercules, CA, USA).

### RNA sample preparation and transcriptome sequencing

The IC50 concentration of Paclitaxel on the Pfeiffer/ADM cells was selected as the concentration for this study. After 48 hours of drug intervention, the total RNA was extracted by the TRIzol solution (Invitrogen, Thermo Fisher Scientific, USA) reagent according to the manufacturer’s instructions. The preparation and sequencing of the RNA library was carried out by Genminix Informatics Company (Shanghai, China).

### Analysis of transcriptome sequencing data

In our study, we analyzed the RNA-seq using the DESeq2 package tool of the R 4.1.0 software. The screening criteria of our differentially expressed genes (DEGs) were set at |log2(FC)|≥1 and adj.*P* ≤ 0.05. To better understand the functions and obtain the annotations of the DEGs and hub genes, the Kyoto Encyclopedia of Gene and Genome(KEGG) pathway analysis and Gene Ontology(GO) functional annotation were performed by utilizing the ClusterProfiler of R Package [22,23].

### Construction of protein–protein interaction (PPI)network and screening of hub genes

The STRING 11.0 database (http://string-db.org/) was used to construct a PPI network between the DEGs and hub genes; the interaction score was set at a high confidence(0.7) [24]. The Cytoscape 3.8.0 software CytoHubba plug-in 6 topology algorithms (Deg, EPC, MNC, MCC, Clo, and BN) were used to screen hub genes [25]. MCODE was used to cluster the PPI network to build the functional modules [26].

### Expression and prognostic analysis of hub genes in public databases

The cBioPortal database [27] (https://www.cbioportal.org/) was utilized to study the mutations of hub genes in DLBCL patients, and the expression of hub genes in DLBCL was evaluated based on the GEPIA2 [28] (http://gepia2.cancer-pku.cn/) and HPA [29] (https://www.proteinatlas.org/) databases. Moreover the relationship between hub genes and clinical stages and overall survival of patients with DLBCL was explored using the UALCA [30] (http://ualcan.path.uab.edu/) database.

### Verification of hub gene expression by real-time reverse transcription PCR (RT-qPCR)

The total RNA extracted from TRIzol was reversely transcribed into cDNA using the PrimeScript™ RT reagent Kitwith gDNA Eraser (TaKaRa, Japan). The SYBR Green Master (ROX) (Thermo Fisher, MA, USA) reagent was used to configure the qPCR system. PCR amplification was performed in the steps (1) 45 seconds at 94°C for denaturation; (2) 45 seconds at 60°C for annealing; and (3) 30 seconds at 72°C for extension, circulating 45 times in the CFX96 Real-Time PCR Detection System. Meanwhile the melting curve of each sample was evaluated in the temperature range of 64–95°C. The relative expression of mRNA was calculated using the 2^−ΔΔ^Ct method, with the context of Glyceraldehyde 3-phosphate dehydrogenase (GAPDH) as the internal reference. The design and synthesis of the primers were entrusted to Sango Biotech (Shanghai, China). The primer sequences are summarized in Table 1.

**Table 1.** Gene primer sequence information

Gene	Forward primer sequence (5’-3’)	Reverse primer sequence (5’-3’)
UBC	GTGTCTAAGTTTCCCCTTTTAAGG	TTGGGAATGCAACAACTTTATTG
TSR1	AAGGAGGCGGTTCTGGCAGAG	TGAGCAAAGAGGCAGGAAAGACAG
WDR46	ATTGTGGAGGCTGTGGACATTGC	GAAGTGGAAGGGCAGGAACTCAAG
HSP90AA1	TCCCGCCCAGAGTGCTGAATAC	GTCTCAACCTCCTCCTCCTCCATC
NOP56	GCCAAGTATCCAGCATCCACAGTG	CGCTTCCTCTGCCTGAACCATTG

## Results

### Comparison of the efficacy of Paclitaxel and Adriamycin in Pfeiffer and Pfeiffer/ADM cells

**Figure 1.**
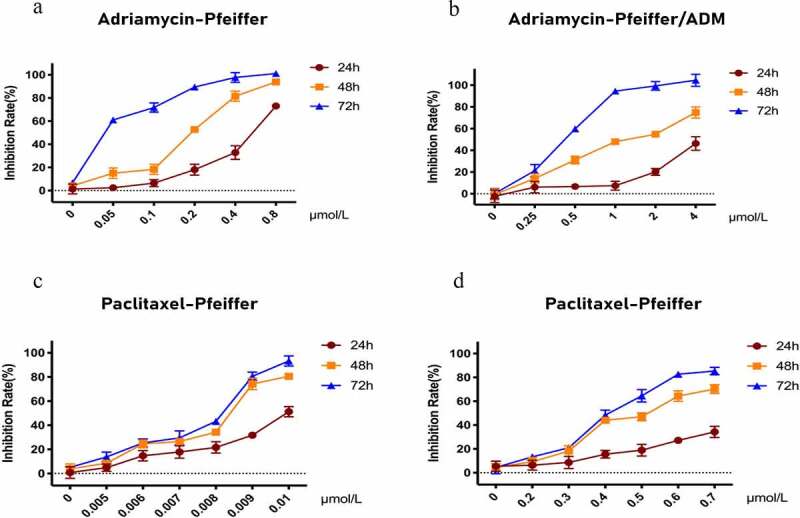
Inhibitory effect of Adriamycin and Paclitaxel on Pfeiffer and Pfeiffer/ADM cells was detected by CCK-8 assays. (a-b) Inhibition rate of Adriamycin on Pfeiffer and Pfeiffer/ADM at 24 h, 48 h, and 72 h. (c-d) Inhibition rate of Paclitaxel on Pfeiffer and Pfeiffer/ADM at 24 h, 48 h, and 72 h. Error bars represent the mean ±SD of triplicate experiments.

**Figure 2.**
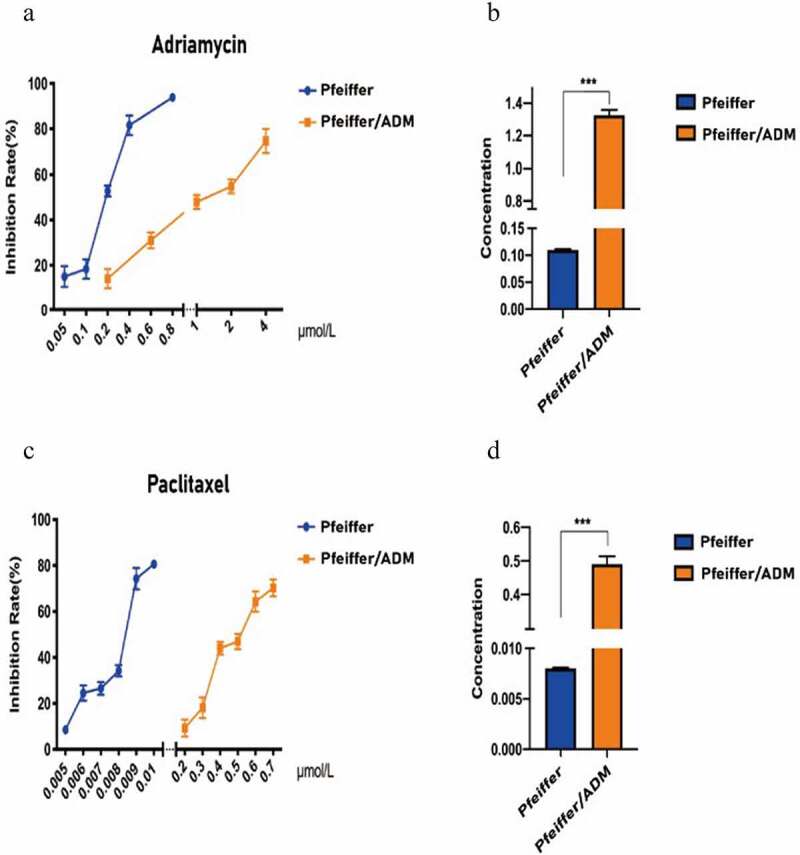
Pfeiffer/ADM cells are more sensitive to Paclitaxel than Adriamycin. (a-b) Calculation and comparison the IC50 of Adriamycin in Pfeiffer and Pfeiffer/ADM cells by plotting the proliferation inhibition rate curve of Adriamycin-treated Pfeiffer and Pfeiffer/ADM cells for 48 hours. (c-d) Calculation and comparion the IC50 of Paclitaxel in Pfeiffer and Pfeiffer/ADM cells by plotting the proliferation inhibition rate curve of Paclitaxel -treated Pfeiffer and Pfeiffer/ADM cells for 48 hours. Error bars represent the mean ±SD of triplicate experiments, compared with Pfeiffer, ***P < 0.005.

The CCK-8 assay results show that Paclitaxel and Adriamycin have proliferation inhibitory effects on both Pfeiffer and Pfeiffer/ADM cells, which are concentration and time dependent (Figure 1). The results showed that the IC50 of Paclitaxel was 0.4912 ± 0.0230 μmol/L in Pfeiffer/ADM, significantly lower than 1.3256 ± 0.0328, which was the value of Adriamycin treatment on Pfeiffer/ADM (*P* ＜ 0.05). This showed that Paclitaxel is more sensitive to Pfeiffer and Pfeiffer/ADM than Adriamycin is (Figure 2 **and** Table 2).

**Table 2.** Comparison of drug resistance between Pfeiffer and Pfeiffer/ADM

Drug	IC50(μmol/L)		Resistance index
	Pfeiffer	Pfeiffer/ADM	
Adriamycin	0.1098 ± 0.0017	1.3256 ± 0.0328***	12.0724
Paclitaxel	0.0080 ± 0.0001	0.4912 ± 0.0230***	61.2862

### DEG identification

**Figure 3.**
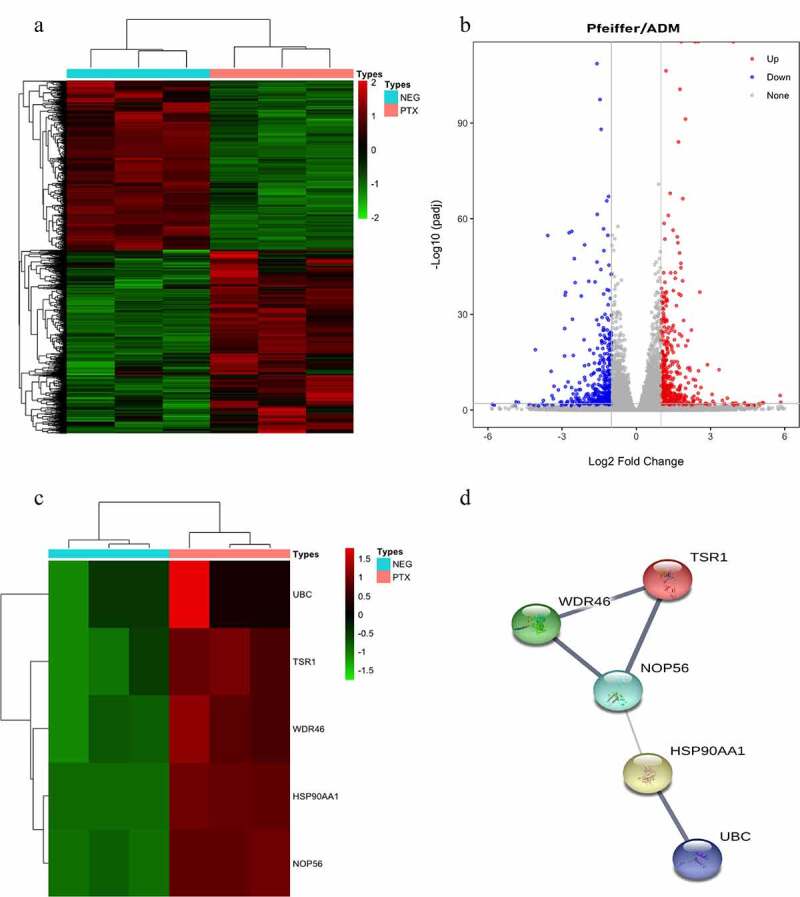
Identification of differentially expressed genes (DEGs) and hub genes in response to Paclitaxel intervention in Pfeiffer/ADM cell lines. (a) Heat map showing 971 DEGs, comparing the control group with the Paclitaxel group. Each row represents one DEG, and each column represents a sample. Red, upregulation; green, downregulation. (b) Volcano plot shows all DEGs identified in the control group and the Paclitaxel group, the 519 red dots represent significantly differentially upregulated genes, and the 452 blue dots represent significantly differentially downregulated genes (log2 (FC)| ≥ 1 and adj. *P* < 0.005). (c) Heat map of the hub genes; (d) PPI networks of the five hub genes.

A total of 971 DEGs were found, including 519 upregulated genes and 452 downregulated genes (Figures 3a and 3b). The genes of the Pfeiffer/ADM negative group (< 1%DMSO) and Paclitaxel group in sequencing data were clustered by R package pheatmap. The expression levels of the Paclitaxel group and negative group genes in Pfeiffer/ADM cells were calculated. The screening criteria for DEGs were as follows: log2 (FC)| ≥ 1 and adj. *P* ≤ 0.05.

### Screening of hub genes

The PPI network map of 971 DEGs (including 907 nodes and 2042 edges) was constructed using the STRING database (Figure 3c). The CytoHubba plug-in of the Cytoscape3.8.0 software was used to screen the hub genes and clusters to construct the functional module. Six algorithms can be used to discover essential proteins of a PPI network. Among them, the test effect of MCC is the most satisfactory. However, to make our research more rigorous, we used the most frequently occurring genes among the six algorithms as hub-genes, and finally the top five genes were used as the hub genes. The results of the CytoHubba analysis showed that UBC, TSR1, WDR46, HSP90AA1, and NOP56 were the hub genes (Table 5); these five hub genes are all significantly upregulated after Paclitaxel acts on Pfeiffer/ADM cells (Figure 3d). The PPI network of the five hub genes showed that there was a highly credible protein interaction between them (Figure 3e **and** Table 6).

**Table 3.** Results of the PPI network clustering function modules for differentially expressed genes

Cluster	Score	Nodes	Edges	Node IDs
1	23.5	25	282	TSR1, RRP12, RRP1, DDX24, POLR1A, MYBBP1A, NOC2L, NOP56, DDX51, RRP1B etc.
2	11.429	36	200	OASL, UBA1, MX1, RPL18A, MEX3C, TRIM36, ASB2, MRPS12, XAF1, RNF19A etc.
3	9	9	36	CCR7, TAS2R43, TAS2R4, NPB, C5, BDKRB2, TAS2R5, CNR1, GRM4
4	8.13	47	187	FABP5, SLC2A8, FBXO5, MSMO1, CDKN1A, NSDHL, SC5D, GLA, DHCR7, GALNS etc.
5	5.481	55	148	NDUFS5, HSPA1B, P4HB, MRPL17, CD3EAP, PFDN6, DYNLL1, PDIA3, NDUFB4, NDUFB3 etc.
6	5	5	10	COPE, KIF27, KDELR1, SURF4, TMED9
7	4	4	6	COL7A1, COL16A1, PPIB, COL14A1
8	4	4	6	OXTR, XCR1, TRHR, NMB
9	4	4	6	HELZ2, MED31, MED1, PPARGC1A
10	4	4	6	NTNG2, CD52, CNTN4, GPLD1
11	3.333	4	5	HSP90AB1, HSPH1, EDF1, STIP1
12	3.333	10	15	CTNNA2, TCAP, TGFB1, TNNI3, TPM2, ACTN4, TGFB3, CTNNA3, MYBPC1, STRAP
13	3	3	3	DCP1A, EIF4A3, RPL7A
14	3	3	3	KDM4A, HIST1H4H, HIST2H3C
15	3	3	3	NELFA, POLR2K, EAF2
16	3	3	3	AHCY, CTH, SDSL
17	3	3	3	GPX1, PRDX5, PRDX1
18	3	3	3	CCDC114, DNAI1, FAM187A
19	3	3	3	MC1R, ADM2, CALCB
20	3	3	3	XYLT2, B4GALT7, SDC3
21	3	3	3	AMT, GCSH, MTHFD2
22	3	3	3	PRIM1, RAD51AP1, CCNB1

**Table 4.** Functional annotation and pathway enrichment analysis of genes in module 1 (TOP10)

ID	Description	Gene Ratio	*P* value	p.adjust	q-value	Genes
**Biological Process**						
GO:0042254	ribosome biogenesis	18/23	3.62E-29	6.95E-27	4.88E-27	TSR1/RRP1/NOC2L/NOP56/DDX51/RRP1B/HEATR1/TBL3/
GO:0022613	ribonucleoprotein complex biogenesis	18/23	7.52E-25	7.22E-23	5.07E-23	TSR1/RRP1/NOC2L/NOP56/DDX51/RRP1B/HEATR1/TBL3/WDR75/EBNA1BP2 etc.
GO:0006364	rRNA processing	15/23	1.27E-24	8.11E-23	5.69E-23	TSR1/RRP1/NOP56/DDX51/RRP1B/HEATR1/TBL3/WDR75/EBNA1BP2/DIMT1 etc.
GO:0016072	rRNA metabolic process	15/23	1.44E-23	6.89E-22	4.84E-22	TSR1/RRP1/NOP56/DDX51/RRP1B/HEATR1/TBL3/WDR75/BNA1BP2/DIMT1 etc.
GO:0034470	ncRNA processing	15/23	1.34E-20	5.16E-19	3.62E-19	TSR1/RRP1/NOP56/DDX51/RRP1B/HEATR1/TBL3/WDR75/EBNA1BP2/DIMT1 etc.
GO:0042273	ribosomal large subunit biogenesis	6/23	2.94E-10	9.40E-09	6.59E-09	NOC2L/EBNA1BP2/PPAN/NIP7/NOP2/FTSJ3
GO:0030490	maturation of SSU-rRNA	5/23	1.74E-09	4.78E-08	3.36E-08	TSR1/HEATR1/TBL3/NAT10/WDR46
GO:0042274	ribosomal small subunit biogenesis	5/23	1.25E-08	3.01E-07	2.11E-07	TSR1/HEATR1/TBL3/NAT10/WDR46
GO:0000462	maturation of SSU-rRNA from tricistronic rRNA transcript (SSU-rRNA, 5.8S rRNA, LSU-rRNA)	4/23	7.26E-08	1.55E-06	1.09E-06	TSR1/HEATR1/TBL3/WDR46
GO:0000154	rRNA modification	4/23	9.28E-08	1.78E-06	1.25E-06	DIMT1/NAT10/NOP2/FTSJ3
**Cellular Component**						
GO:0030684	preribosome	13/25	4.14E-26	1.08E-24	6.53E-25	TSR1/RRP1/NOC2L/NOP56/RRP1B/HEATR1/TBL3/EBNA1BP2/WDR46/PPAN etc.
GO:0030687	preribosome, large subunit precursor	7/25	3.60E-16	4.68E-15	2.84E-15	RRP1/NOC2L/RRP1B/EBNA1BP2/PPAN/NIP7/FTSJ3
GO:0032040	small-subunit processome	5/25	9.22E-10	7.99E-09	4.85E-09	NOP56/HEATR1/TBL3/WDR46/NOL6
GO:0030688	preribosome, small subunit precursor	4/25	1.44E-09	9.38E-09	5.69E-09	TSR1/RRP1/RRP1B/FTSJ3
GO:0030686	90S preribosome	4/25	4.73E-08	2.46E-07	1.49E-07	NOC2L/HEATR1/TBL3/NOL6
GO:0044452	nucleolar part	6/25	8.31E-08	3.60E-07	2.19E-07	POLR1A/NOP56/RRP1B/HEATR1/TBL3/NOL6
GO:0001650	fibrillar center	2/25	0.01249	0.04638	0.02816	NOP56/HEATR1
GO:0005736	RNA polymerase I complex	1/25	0.01516	0.04926	0.02992	POLR1A
**Molecular Function**						
GO:0030515	snoRNA binding	4/25	9.64E-08	4.91E-06	3.55E-06	TSR1/NOP56/HEATR1/TBL3
GO:0008649	rRNA methyltransferase activity	3/25	3.29E-06	5.60E-05	4.04E-05	DIMT1/NOP2/FTSJ3
GO:0140102	catalytic activity, acting on a rRNA	3/25	3.29E-06	5.60E-05	4.04E-05	DIMT1/NOP2/FTSJ3
GO:0140098	catalytic activity, acting on RNA	6/25	7.79E-06	9.94E-05	7.18E-05	DDX24/POLR1A/DIMT1/DDX54/NOP2/FTSJ3
GO:0004386	helicase activity	4/25	5.68E-05	0.00058	0.00042	DDX24/DDX51/DDX55/DDX54
GO:0008173	RNA methyltransferase activity	3/25	9.45E-05	0.0008	0.00058	DIMT1/NOP2/FTSJ3
GO:0008757	S-adenosylmethionine-dependent methyltransferase activity	3/25	0.00133	0.00969	0.007	DIMT1/NOP2/FTSJ3
GO:0003724	RNA helicase activity	2/25	0.00209	0.01334	0.00963	DDX24/DDX54
GO:0008168	methyltransferase activity	3/25	0.00342	0.01937	0.01399	DIMT1/NOP2/FTSJ3
**KEGG pathway**						
hsa03008	Ribosome biogenesis in eukaryotes	6/7	3.03E-11	9.08E-11	3.19E-11	NOP56/HEATR1/TBL3/WDR75/NAT10/NOL6
hsa03020	RNA polymerase	1/7	0.02676	0.04014	0.01409	POLR1A

**Table 5.** Six topological algorithms are used in CytoHubba to screen the TOP 5 hub genes

Category	Rank methods in CytoHubba					
	Degree	MCC	MNC	EPC	Clo	BN
Top 10 genes	UBC	WDR46	UBC	TBL3	UBC	UBC
	NOP56	NOP56	NOP56	WDR46	HSP90AA1	GAPDH
	HSP90AA1	DDX55	HSP90AA1	DDX55	POLR2A	ACTB
	WDR46	TSR1	RPS5	TSR1	ACTB	HSP90AA1
	RPS5	FTSJ3	TBL3	WDR75	HSP90AB1	POLR2A
	TBL3	NOC2L	WDR46	NOP56	GAPDH	HSP90AB1
	POLR2A	GRWD1	POLR2K	FTSJ3	POLR2K	IRF4
	POLR2K	NOP2	TSR1	NOL6	VCP	CCT5
	TSR1	RRP12	RPS13	NOP2	CCT7	EGFR
	POLR1A	DDX54	FTSJ3	HEATR1	CCT5	HSP90B1

Note: Degree (Deg), Edge Percolated Component (EPC), Maximum Neighborhood Component (MNC), Maximal Clique Centrality (MCC), Closeness (Clo), BottleNeck (BN)

**Table 6.** Differential analysis results of the top 5 hub genes in RNA-seq

Gene symbol	log2FoldChange	lfcSE	stat	p-value	p.adj
UBC	1.39	0.307	4.527	5.99E-06	7.02E-05
TSR1	1.033	0.111	9.304	1.35E-20	8.73E-19
WDR46	1.006	0.098	10.231	1.44E-24	1.25E-22
HSP90AA1	2.498	0.063	39.655	0	0
NOP56	1.747	0.07	25.035	2.54E-138	5.06E-135

### Bioinformatics analysis of hub genes in public databases

**Figure 4.**
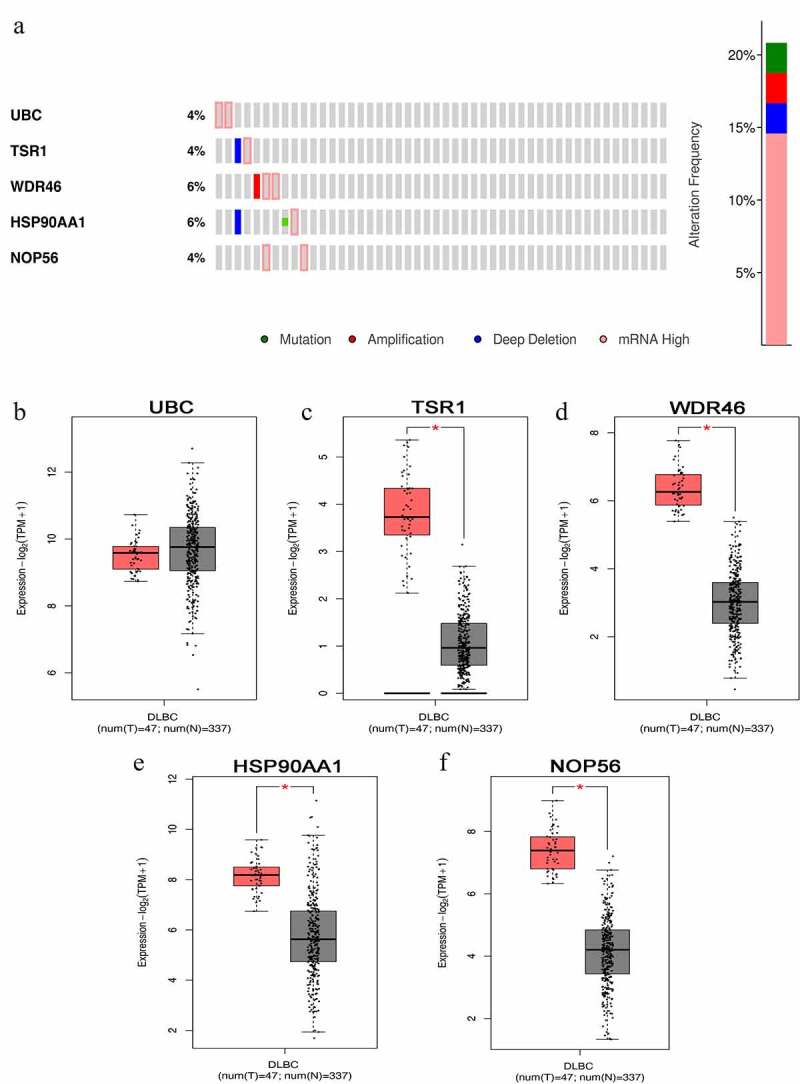
Hub genes mutation and expression analyses in DLBCL (cBioPortal and GEPIA2 databases) (a) Summary of alterations in hub genes in DLBCL. (b-f) Expression of hub genes in DLBCL and normal tissues. (b) UBC; (c) TSR1; (d) WDR46; (e) HSP90AA1; and (f) NOP56. (Red represents DLBCL tissue, gray represents normal tissue, *P < 0.05).

**Figure 5.**
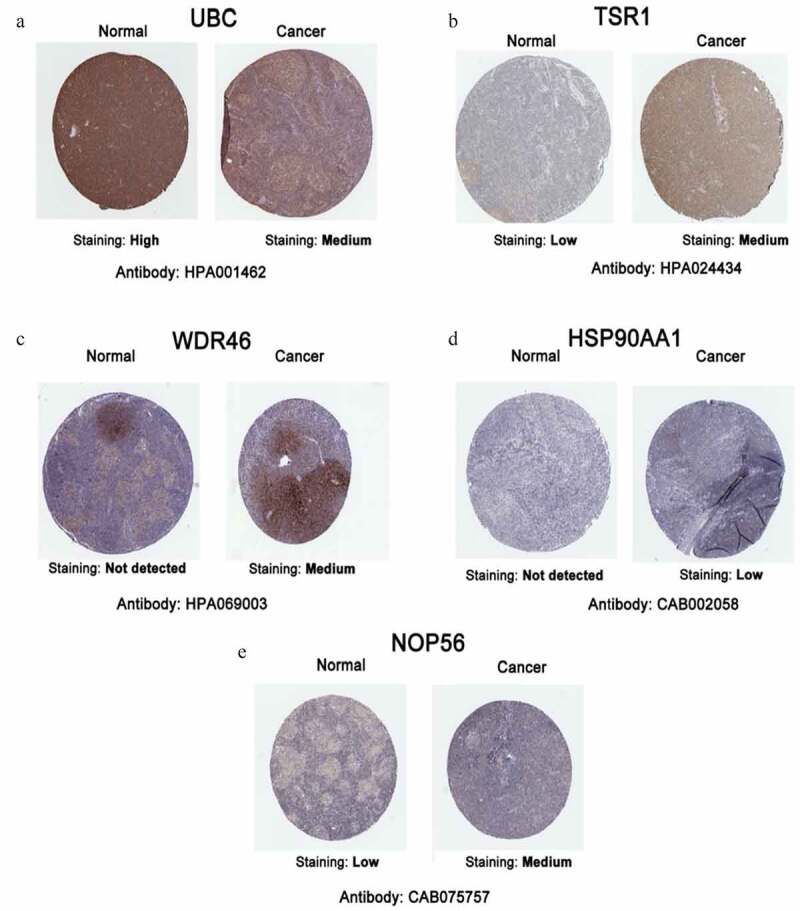
Representative immunohistochemistry images of hub genes in DLBCL and noncancerous lymphoma tissues derived from the HPA database. (a) UBC; (b) TSR1; (c) WDR46; (d) HSP90AA1; and (e) NOP56.

**Figure 6.**
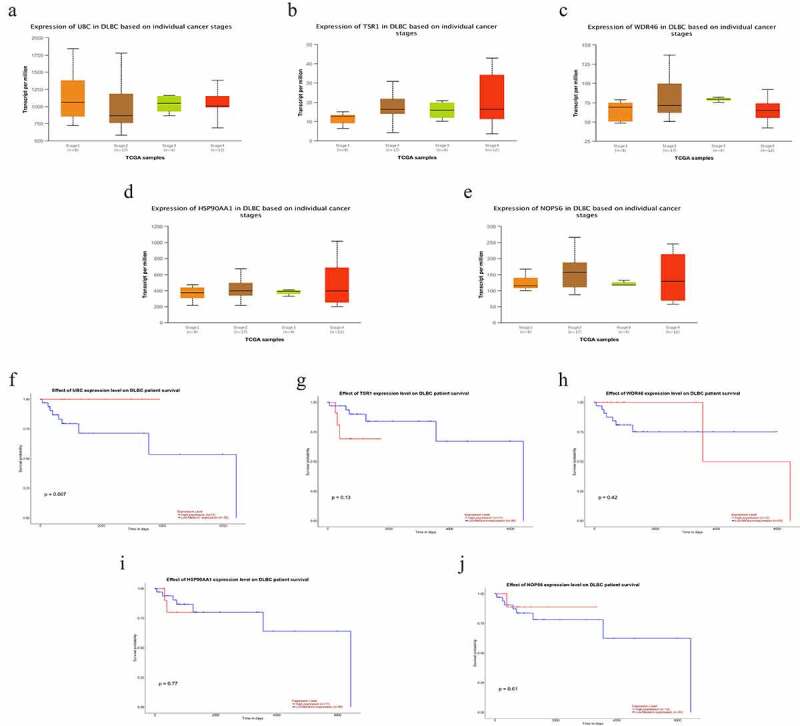
Subgroup expression analyses and survival analyses of hub genes in DLBCL patients using the UALCAN database. (a-e) The mRNA expression levels of hub genes in different clinical subgroups of DLBCL. (a) UBC; (b) TSR1; (c) WDR46; (d) HSP90AA1; and (e) NOP56. (f-j) Relationship between the expression level of hub genes and the overall survival time of DLBCL patients. (f) UBC; (g) TSR1; (h) WDR46; (i) HSP90AA1; and (j) NOP56.

The cBioPortal database showed that WDR46 (6%) and HSP90AA1 (6%) were the two genes with the most genetic changes among the five hub genes (Figure 4a). The mRNA expression of the hub genes in 47 cases of DLBCL and 337 cases of normal tissues was evaluated using the GEPIA2 database. The results showed that the mRNA expression levels of TSR1, WDR46, HSP90AA1, and NOP56 in DLBCL were significantly higher than those in normal tissues (*P* < 0.05), but the expression of UBC was not significant (*P* > 0.05) (Figure 4b and 4f). The HPA database results showed that the protein levels of TSR1, WDR46, HSP90AA1, and NOP56 in lymphoma were higher than those in normal lymphoid tissue, whereas the protein level of UBC in lymphoma was lower than that in normal lymphoid tissue (Figure 5). The results of the UALCAN database showed that the expression of the hub genes TSR1, HSP90AA1, and NOP56 increased gradually with the advancing clinical stage of DLBCL patients, but there was no statistical significance (*P* > 0.05). A survival analysis showed that low expression of UBC and NOP56 represented the trend of risk factors, but there was no statistical significance. The low expression of TSR1 showed a good prognosis trend, but there was no statistical significance (*P* > 0.05) (Figure 6).

### Integrated network analysis

**Figure 7.**
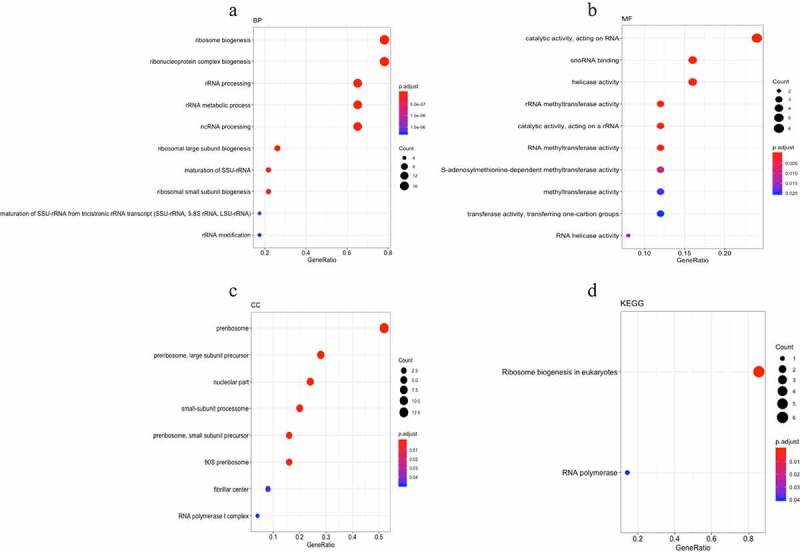
Gene Ontology (GO) and Kyoto Encyclopedia of Genes and Genomes (KEGG) pathway enrichment of genes in module 1. (a-c) The top 10 elements significantly enriched in the GO categories. (a) Biological process; (b) molecular function; and (c) cellular component. (d) 25 genes in model 1 were enriched on two KEGG pathways.

The MCODE plug-in analysis of the PPI network yielded a total of 22 functional modules (Table 3), of which the module ranking first contained 25 nodes and 282 edges (**Supplementary Figure 1**). GO annotation and KEGG pathway enrichment analyses were performed to explore the functions of these 25 genes of module 1 as well as the relevant molecular mechanism (Table 4). The enriched GO terms for the biological process (BP) of upregulated DEGs included ribosome biogenesis, ribonucleoprotein complex biogenesis, and rRNA processing. Moreover, enriched GO terms for the cellular component (CC) also revealed that upregulated DEGs were mainly involved in the preribosome, 11 large subunit precursors, and small-subunit processome. Furthermore, enriched GO terms for the molecular function (MF) were mainly enriched in snoRNA binding, rRNA methyltransferase activity, catalytic activity, and acting on a rRNA. The KEGG pathway enrichment analysis revealed that the upregulated DEGs were mostly enriched in terms of ribosome biogenesis in eukaryotes and RNA polymerase (Figure 7 **and** Table 4). -

### The results verified by RT-qPCR

**Figure 8.**
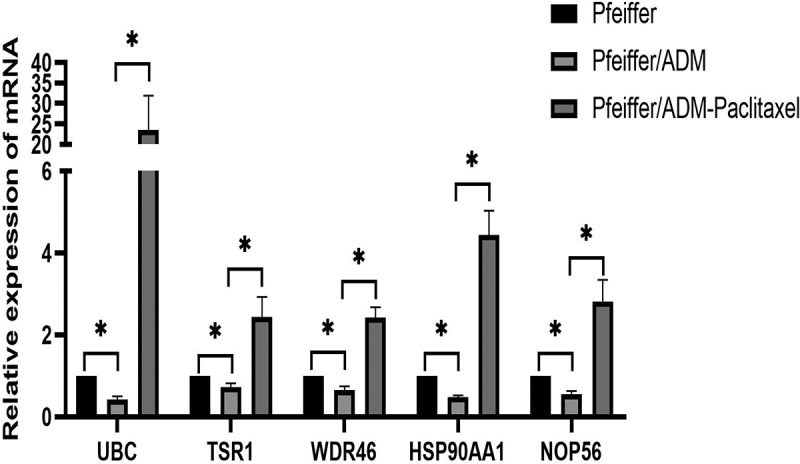
The relative expression of mRNA of five hub genes among the Pfeiffer group, Pfeiffer/ADM group, and Pfeiffer/ADM Paclitaxel-treated group were determined by RT-qPCR assays. The Pfeiffer group was regarded as the control group and normalized; error bars represent the mean ± SD of triplicate experiments, *P < 0.005.

The relative mRNA expression of mRNA of five hub genes among the Pfeiffer group, Pfeiffer/ADM group, and Pfeiffer/ADM Paclitaxel-treated group is displayed in Table 7. The mRNA levels of the five hub genes in the Pfeiffer/ADM group were significantly lower than those in the Pfeiffer group and the Pfeiffer/ADM Paclitaxel-treated group. and the difference was statistically significant (*P* < 0.05) (Figure 8). The experimental results of the RT-qPCR are consistent with the sequencing results, and the reliability of RNA-seq can be confirmed.

**Table 7.** Relative mRNA expression levels of hub genes among Pfeiffer group, Pfeiffer/ADM group, and Pfeiffer/ADM Paclitaxel-treated group

Gene	Relative expression		
	Pfeiffer group	Pfeiffer/ADM group	Pfeiffer/ADM Paclitaxel-treated group
UBC	1.00	0.431 ± 0.071	23.481 ± 83,438
TSR1	1.00	0.727 ± 0.091	2.441 ± 0.482
WDR46	1.00	0.650 ± 0.094	2.421 ± 0.255
HSP90AA1	1.00	0.477 ± 0.046	4.444 ± 0.591
NOP56	1.00	0.552 ± 0.077	2.819 ± 0.522

## Discussion

Paclitaxel or Paclitaxel combined with conventional chemotherapeutic drugs was found to be effective in the treatment of Adriamycin-resistant DLBCL patients [31]. Similarly, foundational studies have also revealed that Adriamycin-resistant cell lines are sensitive to Paclitaxel and its derivative docetaxel, which can greatly improve the inhibition rate of these cell lines after the action of the drugs [18]. In our study, we screened and obtained five hub genes and one important gene module based on the analysis of RNA-seq data of Paclitaxel-treated Adriamycin -resistant DLBCL cells. The bioinformatics analysis showed that WDR46 (6%) and HSP90AA1 (6%) were the two genes with the highest level of genetic changes. The expression levels of mRNA and proteins of TSR1, WDR46, HSP90AA1, and NOP56 in DLBCL were significantly higher than those in normal tissues, suggesting that these four hub genes can be activated and can upregulate the expression of mRNA during tumor development. The gene expression of TSR1, HSP90AA1, and NOP56 gradually increased with the advancement of the clinical stage.

In the field of tumor research, the expression of UBC is closely related to the progression of tumors. Melatonin inhibits endometrial cancer progression by inhibiting succinate accumulation induced by the estrogen/UBC/SDHB signaling pathway [32]. Simultaneous knockdown of UBB and UBC mRNAs induces gastric cancer cell apoptosis, resulting in decreased cell viability, thereby inhibiting gastric cancer cell metastasis [33]. The expression of HSP90AA1 is closely related to tumor proliferation, apoptosis, and the development of drug resistance. A high expression of HSP90AA1 can increase the chemical resistance of ovarian cancer SKOV3 cells to cisplatin and reduce the apoptosis induced by cisplatin [34]. As an important regulator of autophagy, HSP90AA1 increases drug resistance by inducing autophagy and inhibiting apoptosis and provides a new therapeutic target for improving the treatment of osteosarcoma [35]. Studies have shown that NOP56 expression is associated with Burkitt’s lymphoma and can be used as a molecular marker for its diagnosis [36], which was also found to be upregulated in diffuse B-cell lymphoma and chronic lymphocytic leukemia [37,38]. Silencing the expression of NOP56 in rectal cancer cells can reduce the proliferation ability of tumor cells [39]. Currently, there is no related research on TSR1 and WDR46 in tumors. The specific molecular mechanism of these five hub genes in Adriamycin -resistant DLBCL has not yet been elucidated. which provides a novel direction for future research. Interestingly, our RT-qPCR results showed that five hub-genes were significantly downregulated in the Adriamycin-resistant cell line Pfeiffer/ADM compared with the Pfeiffer cell line, but Paclitaxel reversed the downregulation trend of five hub-genes, and significantly upregulated them. We speculate that these five hub genes play an important role in the process of doxorubicin resistance. The downregulation of hub genes may be related to the resistance of Pfeiffer cell line, and paclitaxel can reverse this epigenetic change.

Functional annotation and pathway analysis showed that ribosome biogenesis has an important role in inhibition of Paclitaxel working on Adriamycin-resistant DLBCL. Ribosome synthesis has recently become an effective target in cancer therapy, whereby compounds that inhibit ribosome production or related functions and give priority to killing cancer cells have been introduced in clinical trials [40]. The latest research showed that ribosome synthesis also plays a key role in tumorigenesis [41]. Ribosomal biosynthetic factor is a clinical marker of acute myeloid leukemia, where ribosome production of the ribosomal nucleoside regulator is overexpressed in acute myeloid leukemia. Moreover, high NCL mRNA expression levels are associated with poor overall survival [42]. Compared with conventional chemotherapy, therapy targeting key ribosome biogenesis can reduce the genotoxic activity of cancer cells. The use of targeted ribosome biosynthesis inhibitors in ovarian cancer, melanoma, and leukemia models with active or mutated p53 status can inhibit cell-dependent activity. This is expected to solve the mechanism of tumor resistance to conventional chemotherapeutic drugs in p53 mutation [43]. Anthracycline-based polychemotherapy is widely used in hematological tumors. With the cardiotoxicity of anthracyclines, the development of selectively targeted ribosome biosynthesis is an urgent clinical problem [44]. There are some limitations of this research, the biological function of hub genes in Adriamycin -resistant cell line Pfeiffer/ADM and the specific molecular mechanism of Paclitaxel against Pfeiffer/ADM still lack further experimental support. Fortunately, CRISPR-screening, a new technology of whole-genome knockout combined with next-generation sequencing, it can elucidate the relationship between genes and phenotypes in high-throughput sequencing, and is widely used in the regulation of cancer drug resistance factor screening [45], thus providing new ideas for our next research.

## Conclusions

The results of this study indicate that Paclitaxel has a strong inhibitory effect on DLBCL and Adriamycin-resistant Pfeiffer/ADM cells, and hub genes (UBC, TSR1, WDR46, HSP90AA1, and NOP56) and ribosome biosynthesis can play a key role in Paclitaxel-induced Pfeiffer/ADM and an important potential role in the future application of Paclitaxel inhibition on Adriamycin-resistant DLBCL. However, there are some limitations of this research, the specific molecular mechanism of Paclitaxel against Pfeiffer/ADM still lack further experimental support.

## Supplementary Material

Supplemental Material
