# Supplementary figures and images for: RNA-seq and integrated network analysis reveals the hub genes and key pathway of paclitaxel inhibition on Adriamycin resistant diffuse large B cell lymphoma cells
Source: Bioengineered. 2022 Mar 9;13(3):7607–21. doi: 10.1080/21655979.2022.2048772 (PMC8973673; doi:10.1080/21655979.2022.2048772)

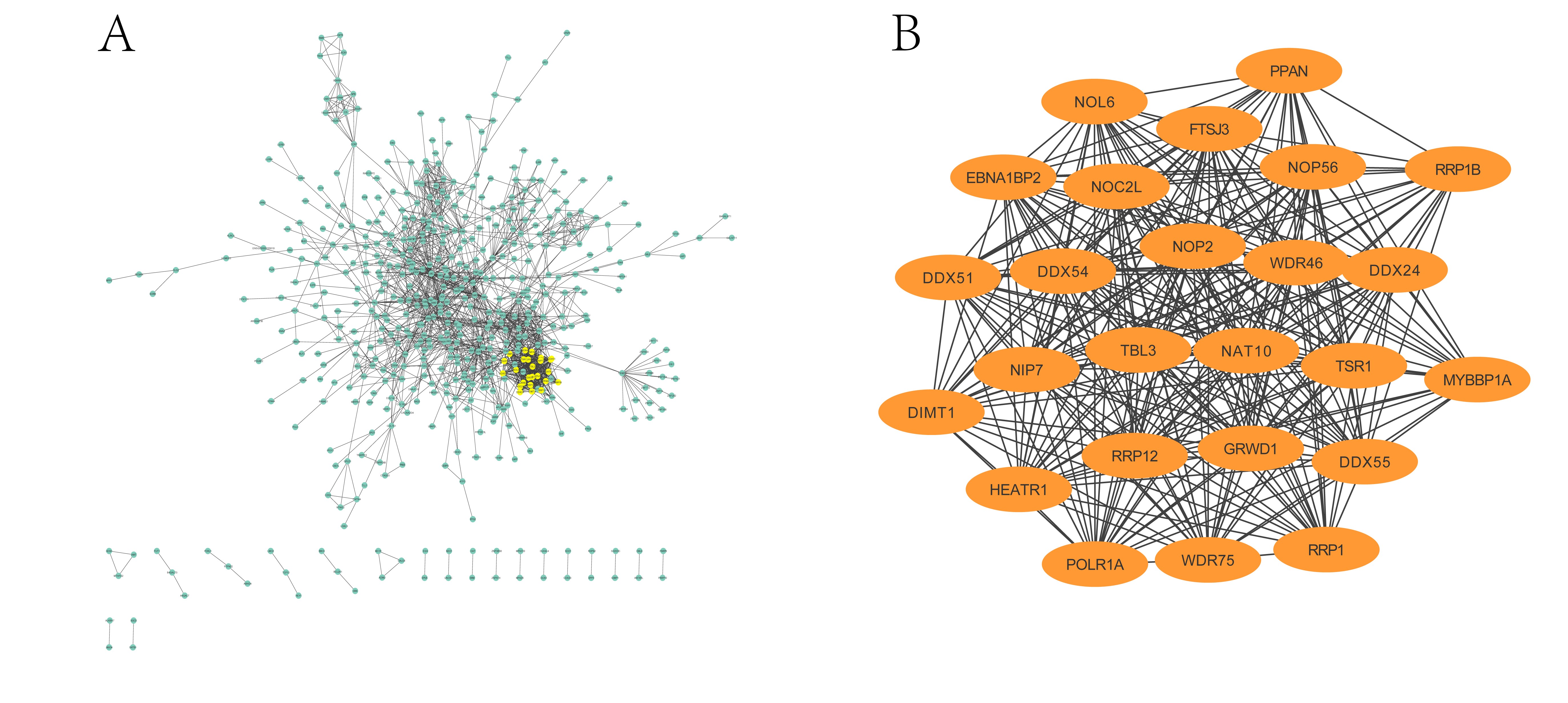

Supplement: Supplemental Material [file KBIE_A_2048772_SM8939.jpg]
